# Oral Contraceptive Use and Breast Cancer Risk According to Molecular Subtypes Status: A Systematic Review and Meta-Analysis of Case-Control Studies

**DOI:** 10.3390/cancers14030574

**Published:** 2022-01-23

**Authors:** Agnieszka Barańska, Joanna Dolar-Szczasny, Wiesław Kanadys, Wiktoria Kinik, Dorota Ceglarska, Urszula Religioni, Robert Rejdak

**Affiliations:** 1 Department of Medical Informatics and Statistics with E-Learning Lab, Medical University of Lublin, 20-090 Lublin, Poland; 2 Department of General and Pediatric Ophtalmology, Medical University of Lublin, 20-070 Lublin, Poland; joannaszczasny@op.pl (J.D.-S.); robert.rejdak@umlub.pl (R.R.); 3 Specialistic Medical Center Czechow, 20-848 Lublin, Poland; wieslaw.kanadys@wp.pl; 4 Science Popularization Centre, Medical University of Lublin, 20-059 Lublin, Poland; wiktoriakinik@umlub.pl; 5 Subunit, Primary Health Care Center Provita, 20-093 Lublin, Poland; nzozprovita@neostrada.pl; 6 School of Public Health, Centre of Postgraduate Medical Education of Warsaw, 01-813 Warsaw, Poland; urszula.religioni@cmkp.edu.pl; 7 National Institute of Public Health-National Institute of Hygiene, Warsaw School of Economics, 02-554 Warsaw, Poland

**Keywords:** oral contraceptives, breast cancer, molecular subtypes status, risk factors, estrogen receptor, progesterone receptor, human epidermal growth factor receptor 2

## Abstract

**Simple Summary:**

Breast cancer (BrCa) is a heterogeneous disease with multiple intrinsic tumor subtypes evidenced by the joint expression of molecular tumor markers. Data from epidemiologic studies provide evidence supporting differential effects of oral contraceptives on risk of developing the distinct subtypes of breast cancer; while some studies suggest increased risk, others show its lack. Toward this objective, we conducted meta-analysis of case-control trials devoted to this topic. The results of our study suggest that the oral contraceptive use has different effects on the risk of developing the various molecular breast cancer subtypes.

**Abstract:**

We conducted a systematic review and meta-analysis to investigate the effect of oral contraceptives (OCs) on risk of breast cancer (BrCa) by status of estrogen receptor (ER), progesterone receptor (PR), and human epidermal growth factor receptor 2 (HER2). We searched the MEDLINE (PubMed), Embase and the Cochrane Library database and bibliographies of pertinent articles published up to 2020. Therein, we identified nineteen eligible case-control studies which provided data by breast cancer subtypes: ER-positive (ER+), ER-negative (ER−), HER2-positive (HER2+) and Triplet-negative (TN). Summary risk estimates (pooled OR [pOR]) and 95% confidence intervals (CIs) were calculated using fixed/random effects models. The summary meta-analysis showed that over-use of OCs led to significant increased risk of TNBrCa (OR = 1.37, 95% CI; 1.13 to 1.67, *p* = 0.002), as well as of ER−BrCa (OR = 1.20, 95% CI: 1.03 to 1.40, *p* = 0.019). There was also a significant reduction in the risk of ER+BrCa (OR = O.92, 95% CI: 0.86 to 0.99, *p* = 0.026,) and a slight reduction in the risk of HER2+BrCa (OR = 0.95, 95% CI; 0.79 to 1.14, *p* = 0.561) after taking OCs. Meta-analysis indicated that OC use has different impacts on risk of breast cancer subtypes defined by receptor status. The identified differences between individual subtypes of breast cancer may reflect different mechanisms of carcinogenesis.

## 1. Introduction

Breast cancer (BrCa) is the most commonly diagnosed malignant neoplasm in women, most often originating in the epithelial tissue of the mammary gland. In 2020, 2,261,419 new cases were estimated, accounting for 11.7% of all cancer cases, and causing 684,996 deaths worldwide. BrCa death rates were significantly higher in developed countries compared to developing countries (15.0 vs. 12.8 per 100,000) [1,2]. Most cases of BrCa are sporadic, but an estimated 5–10% have a genetic predisposition related to, among other things, a family history of cancer in first-degree relatives or carrying genetic mutations [3,4].

BrCa is a heterogeneous disease with multiple intrinsic tumor subtypes evidenced by the joint expression of molecular tumor markers such as estrogen receptor (ER), progesterone receptor (PgR), human epidermal growth factor 2 (HER2, ERBB2) and a proliferation index (Ki67), based on their presence or absence; together with tumor size, tumor grade and nodal status [5]. Subtypes differ in their genomic and immunohistochemical signatures, distinct racial/ethnic-specific incidence patterns and varying degrees of aggressiveness, and this is associated with worse treatment responses and prognosis [6].

The most common group of BrCa is ER-positive (ER+). This includes the two subgroups, luminal A and luminal B, occurring in about 60% of all cancers [7,8]. The luminal A (ER+/PgR+/HER2− with low Ki67) subtype comprises about 40% of all cases, and is characterized by slow growth, low aggressiveness, low relapses, high survival rate, and the best prognosis and response to hormone therapy [9]. In turn, the luminal B (ER+/PgR+/HER2+ or HER2− with high Ki67) subtype is responsible for 10–20% of all cancer cases, has higher relapse rate, histological grade, proliferative index, and the lower relapse survival rate [10,11,12]. 

The distinct group of BrCa is of the ER-negative (ER−) subtype. Its characteristic morphological features are: infiltrative margin, high grade, lymphoid stroma, central fibrosis/necrosis, and comedo-type necrosis. It is worth pointing out that the most invasive cancers (20–25%) are of the ER−/PgR− subgroup associated with a lower endocrine therapy sensitivity score [13], and some cancers show a higher BRCA 1 germline mutation [14].

There are more and more reports showing the existence of a unique BrCa subtype with an ER−/PgR+ phenotype (about 3%), with different molecular and clinical characteristics. This subtype is characterized by a low result of hormone sensitivity and a tendency of early relapse and worse overall survival [15,16,17]. However, many researchers question the existence of this subtype, arguing that it is not reproducible, biologically unlikely, or is a technical artifact dependent on an immunohistochemical procedure leading to misclassification [18,19,20,21].

HER2-positive BrCa, defined as ER−/PgR−/HER2+, accounts for 10% to 34% of all invasive breast cancers. It stands out because of its tendency to grow and spread more aggressively. It is associated with shorter disease-free status and overall survival [22,23,24]. Triple-negative breast cancer (TNBrCa) (ER−/PgR−/HER2−) preferentially affects young women and accounts for 12–17% of all breast cancers. This is one of the more aggressive cancers, and is characterized by high mortality and risk of metastasis [25,26].

Although differences in the etiology of the respective breast cancer subtypes are not fully understood, there are many risk factors, among others: reproductive, genetic, lifestyle, BrCa in family history, carrier of the mutation, age of menarche, parity, age at first birth, breastfeeding, or exogenous hormone use [27,28,29]. In addition, the role of estrogens in the etiology of BrCa is significant by stimulating growth and proliferation of ductal epithelial cells in the breast; thus, the status of the estrogen receptor in breast carcinomas provides one of the earliest research objects [30,31].

The relationship between oral contraceptive (OC) taking and the risk of breast cancer has also been extensively researched. Findings suggest that OC use is associated with a moderately increased breast cancer risk in the general population [32,33,34,35].

Data from epidemiologic studies provide evidence supporting differential effects of oral contraceptives on risk of developing the distinct subtypes of breast cancer; while some studies suggest increased risk, others show its lack. Toward this objective, we conducted meta-analysis of case-control trials devoted to this topic.

## 2. Materials and Methods

### 2.1. Search Strategy and Selection Criteria

This systematic review with meta-analysis was designed according to PRISMA (Preferred Reporting Items for Systematic Reviews and Meta-Analyses) guidelines [36,37] to determine if use of oral contraception, as compared to placebo, affects the risk of the breast cancer subtypes (Supplementary File S1—PRISMA 2020 Checklist). The bibliographic databases MEDLINE (PubMed), Embase and the Cochrane Library were searched for the identification of case-control studies that were conducted up to June 2020. The following search terms were used for all databases in various combinations: “oral contraceptives” or “birth control pill” AND “subtype breast cancer risk” or “ER+ subtype” or “ER− subtype” or “HER2 positive” or “TNBrCa”. References of found articles, previous review articles and meta-analysis, and other relevant publications related to the topic of the work were also searched in order to identify further pertinent studies. Articles were initially evaluated according to title and/or abstract. Next, the decision was made to include or exclude after independent and double analysis, and full tests of selected studies. Relevant research data was extracted from the full-text works selected for inclusion.

We included publications written in English, based on case-control studies (population- and hospital-design), providing information about the association between oral contraceptive use and breast cancer by ER, PR or HER2 status, and the data contained therein were sufficient to calculate the odds ratio (OR) and the 95% confidence interval (CI). The exclusion criteria were as follows: insufficient data for calculating desired parameters, the results were reported as graphics; duplicate reports; and reviews or case only studies.

### 2.2. Data Extraction

The following data were extracted for each study: (i) clinical and methodological study characteristics such as last name of first author, publication year, country in origin, years of data collection, number of participants in case and control subgroups; (ii) information on use of OC in individual subgroups: whenever/never, duration, age at first use, and years since last use prior to diagnosis; (iii) in the original studies, different definitions and combinations of subgroup used; in our analysis, we grouped subtypes into the following four categories: ER-positive (regardless of their PR/HER2 status), ER-negative (regardless of their PR status), HER2-positive (absence of ER/PR), and Triplet-negative (absence of ER/PR/HER2).

### 2.3. Assessment of Study Quality

The Newcastle–Ottawa Scale (NOS) score was employed to evaluate methodological quality of included studies. With this tool, each study was assessed in three separate categories: selection of cases and controls, comparability of cases and controls on the basis of the design or analysis, and ascertainment of exposure. A maximum score was 9, of which 0–3, 4–6, and 7–9 scores were considered as low, fair, and high quality [38].

### 2.4. Statistical Analysis

The distribution of cases and controls at risk, ORs and 95% CI were separately identified by receptor status and for oral contraceptive use (ever or never) and by age of first use of OCs, duration of OC use, and years since last use of OCs prior to diagnosis, when data were available. We calculated the summary risk estimates and 95% CIs and plotted forest plots using random-effects models (DerSimonian–Laird method) for the association between ever oral contraceptives use and breast cancer by receptor status. The results indicated that the taking of OCs may have a high probability of increase in risk if OR was above 1, compared with non-use of OCs [39].

Heterogeneity among articles was estimated by engaging the I^2^ statistic and *p* values associated with Q statistics. I^2^ statistic indicates the percentage of total variability explained by heterogeneity, and values of ≤25%, ‘25%–75%’, and ≥75% are arbitrarily considered as indicative of low, moderate, and high heterogeneity, respectively [40].

To explain the possible presence of publication bias, Begg’s test (a rank correlation method based on Kendall’s tau) and Egger’s test (a linear regression method) were applied [41,42]. We also checked for funnel plot symmetry. Here, in the absence of bias, the plots will resemble a symmetrical funnel, as the results of minor studies will scatter at the left side of the plot and the spread will narrow among the major studies on the right side of the plot [43]. In order to explain the possible influence of covariates such as age at first OC use (<25 years/≥25 years), duration of OC taking (≥5 years/<5 years), and years since last OC use prior to diagnosis (<5/≥ 5) on risk of individual of breast cancer subtypes, we performed a meta-regression [44]. Meta-analysis of summary statistics from individual studies was performed through Statistica 13.3 software (StatSoft Poland, Kraków, Poland), using the Medical Package program.

## 3. Results

**Figure 1:**
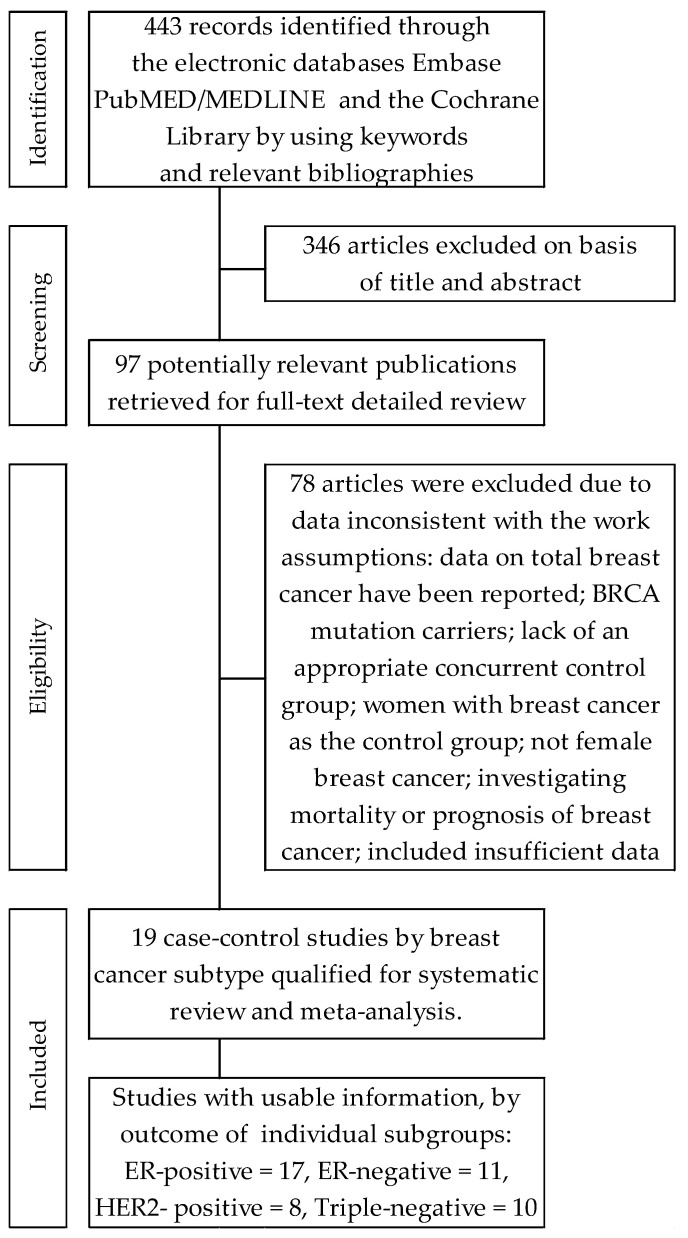
Flowchart of the selection procedure for studies included in the current review and meta-analysis.

Searches of electronic databases allowed the identification of four hundred and forty-three (443) citations. Subsequently, three hundred forty-six (346) items were excluded on the basis of title and/or abstracts. In turn, ninety-seven (97) articles with potentially significant case-control works were identified and submitted for full-text assessment. Of these, seventy-eight (78) papers contained duplicate publications, or included insufficient data for calculating the desired parameters, or for other reasons, did not meet all the inclusion criteria. Finally, nineteen articles were included in the systematic review and meta-analysis. A detailed review of selection procedure is shown in Figure 1.

The quality of the analyzed studies as assessed on the basis of the Newcastle–Ottawa Scale (NOS) ranged between 4–8, and the average score was 6.74 for included studies. Furthermore, 12 (63.16%) were considered high quality studies (NOS ≥ 7 points).

Thirteen (13) case-control studies were conducted in North, three (3) in Asia, and one each in Australia, and Europe, while one pooled study was conducted in the United States, Canada, Australia and Korea. The studies involved a total of 246,152 persons, including 31,250 cases of breast cancer and 214,902 people as control. Six studies exclusively included premenopausal women [45,46,47,48,49,50].

The present meta-analysis was conducted on the basis of data from nineteen case-control studies assessing the effect of oral contraceptives on the risk of individual molecular subtypes of BrCa. Characteristics of selected works are shown in Table 1. 

### 3.1. Effects of Oral Contraceptive Use on ER-Positive Breast Cancer 

**Figure 2:**
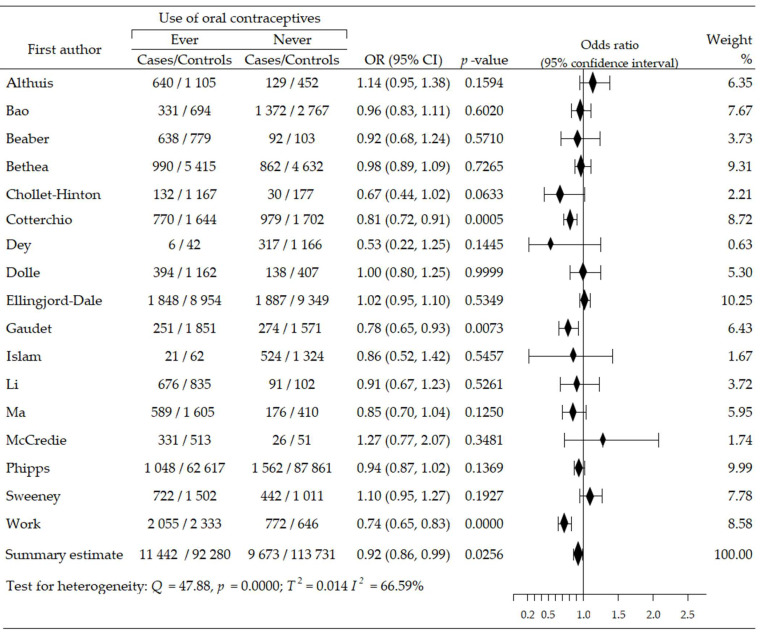
Forest plot and summary odds ratios on the association between risk of ER+BrCa and ever-use of oral contraceptives. Note: black diamonds represent the effect sizes; the horizontal lines denote the 95% confidence interval.

Seventeen trials [46,47,48,49,50,51,52,53,54,55,56,57,58,59,60,61,62,63] contributed to the meta-analysis on the effects of oral contraceptives use on the acquisition risk of ER-positive breast cancer (ER+BrCa). Here, the data were pooled from studies comprising a total of 227,126 women, including 21,115 and 206,126 participants in the intervention and control arms, respectively. These studies included four that reported a non-significant increase in the risk of ER+BrCa [46,47,52,61]; in the remaining studies, a risk reduction was noted, including three wherein the changes were statistically significant [51,55,59]. The summary meta-analysis showed that ever-use of OC significantly decreased ER+BrCa risk, compared with never-use: OR = O.92, 95% CI: 0.86 to 0.99, *p* = 0.026, I^2^ = 66.59% (Figure 2). No significant publication bias was found after Begg’s (*p* = 0.529) and Egger’s (*p* = 0.384) test assessments (Table 2).

Analysis based on data from three studies [54,57,61] did not reveal the significant effect of age at first use of OCs on ER+BrCa risks for <25 years (OR = 0.93, 95% CI: 0.72 to 1.19, *p* = 0.550, I^2^ = 90.67%); or for ≥ 25 years (OR = 0.98, 95% CI: 0.92 to 1.04, *p* = 0.470, I^2^ = 0.00%). The Begg’s test indicated no evidence of publication bias for age ≥ 25 years (*p* = 0.117); the result for age < 25 years was inaccessible, whereas the Egger’s test reported lack of publication bias for age < 25 years (*p* = 0.305); however, the result of the test was significant (*p* = 0.008) for age ≥ 25 years [Table 2]. Multivariable meta-regression with covariate of age of participants showed that this covariate had no significant influence: β = 0.03, 95% CI: −0.21 to 0.28, *p* = 0.798. 

In turn, a review of ten studies [46,48,49,51,52,54,57,59,60,61] revealed that OC use longer than five years leads to non-significant decrease in ER+BrCa risk (OR = 0.91, 95% CI: 0.82 to 1.02, *p* = 0.124, I^2^ = 82.41%); and use of OC for less than five years lowered the risk of cancer. This last result was statistically significant: OR = 0.93, 95% CI: 0.87 to 1.00, *p* = 0.042, I^2^ = 53.09%. A comparison of OC use for more than five years and less than five years showed that the risk of developing ER+BrCa was similar: OR = 0.99, 95% CI: 0.91 to 1.08, *p* = 0.791. No evidence of publication bias was recorded for the duration of OC use, according to Begg’s and Egger’s tests (Table 2). Meta-regression with the covariates of duration of OC use demonstrated lack of influence on ER+Ca subtype risk: β = 0.02, 95% CI: −0.12 to 0.15, *p* = 0.815.

We also assessed the relationship between ER+BrCa risk and years since last OCs use prior to diagnosis, based on five studies [46,48,52,54,60]. These revealed that the last use of OC in the period less than five years before diagnosis was associated with a non-significant reduction in subtype risk: OR = 0.94, 95% CI: 0.77 to 1.15, *p* = 0.555, I^2^ = 66.34%. In turn, the last use of OC ≥ 5 years before diagnosis resulted in a marginal increase in cancer risk: OR = 1.05, 95% CI: 0.95 to 1.17, *p* = 0.346, I^2^ = 49.58%. The Begg’s and Egger’s tests showed no publication bias for the last OCs use prior to diagnosis of this breast cancer, *p* = 0.693 and *p* = 0.798, respectively (Table 2). Moreover, multivariable meta-regression with covariate of years since last OCs use prior to diagnosis showed no significant influence on risk of ER+BrCa subtype: β = 0.03, 95% CI: −0.21 to 0.28, *p* = 0.798. 

Finally, we examined whether the risk of developing ER+BrCa differs between premenopausal and postmenopausal women. It turned out that the risk was increased in premenopausal women, but the results were not statistically significant: OR = 1.07, 95% CI: 0.85 to 1.34, *p* = 0.575. No significant publication bias was detected by the Egger’s test (*p* = 0.3752).

### 3.2. Effects of Oral Contraceptive Use on ER-Negative Breast Cancer

**Figure 3:**
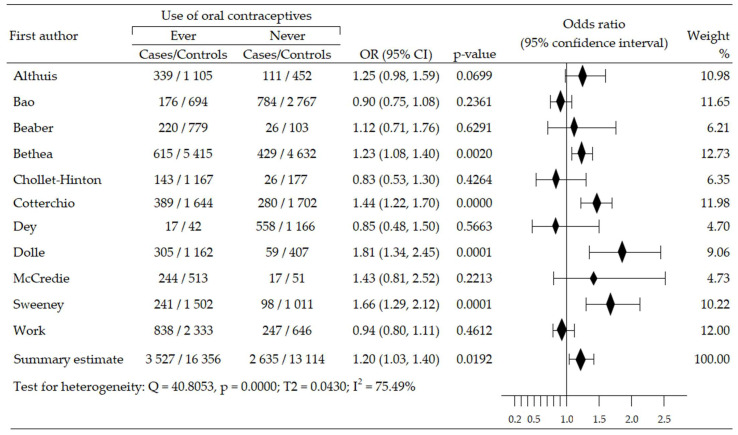
Forest plot and summary odds ratios on the association between risk of ER−BrCa and ever-use of oral contraceptives. Note: black diamonds represent the effect sizes; the horizontal lines denote the 95% confidence interval.

The relationship between the use of oral contraceptives and the risk of ER-negative breast cancer (ER−BrCa) subtype was assessed on the basis of 11 studies [46,47,48,50,51,52,53,56,59,60,62]. These studies involved 35,632 women participants (cases group—6162, control group—29,470). Increased risk of cancer was observed in seven studies [46,47,48,50,51,52,60], including four wherein the increase in risk was statistically significant [48,51,52,60]; in four studies, the risk reduction was insignificant [53,56,59,62]. Compared to non-users of OC, the summary meta-analysis showed that ever-use of OC brought about a significant increase of ER−BrCa risk: OR = 1.20, 95% CI: 1.03 to 1.40, *p* = 0.019, I^2^ = 75.49% (Figure 3). The results of Begg’s (*p* = 0.929) and Egger’s (*p* = 0.927) tests indicate the lack of evidence of publication bias (Table 2). 

Beyond the aforementioned, six studies [48,51,52,56,59,60] rated the effect of duration of OC taking of OC on ER−BrCa subtype risk. OC use for a period ≥ 5 years showed a non-significant increase in risk: OR = 1.19, 95% CI: 0.81 to 1.76, *p* = 0.373, I^2^ = 94.39%. In addition, OC self-administration for <5 years induced a non-significant risk of this cancer subtype developing: OR = 1.14, 95% CI: 0.93 to 1.40, *p* = 0.201, I^2^ = 79.46. On the other hand, the comparison of these two groups showed a significantly higher risk of ER−BrCa in the case of OC use for more than five years: OR = 1.14, 95% CI: 1.01 to 1.27, *p* = 0.031. Results of Begg’s test were inaccessible for variable ≥ 5 years, while that for the covariate < 5 years suggested the possibility of a publication bias (*p* = 0.041). In turn, Egger’s test did not indicate publication bias for both covariates, ≥5 years (*p* = 0.230) and <5 years (*p* = 0.436). When comparing these groups, both the Begg’s (*p* = 0.005) and Egger’s tests (*p* = 0.000) showed the possibility of a publication bias; however, all publications in the funnel plot were placed inside the funnel (Table 2). Moreover, the results of multivariable meta-regression for the covariates of the duration of OC use demonstrated that they did not have a significant effect on this subtype: β = −0.17, 95% CI: −0.46 to 0.11, z = −1.17, *p* = 0.241. 

The meta-analysis of the risk of ER−BrCa subtype depending on the period of discontinuation taking of OC before diagnosis was based on four studies [46,48,52,60]. It showed a statistically significant increase for variable < 5 years (OR = 1.77, 95% CI: 1.35 to 2.32, *p* = 0.000, I^2^ = 68.44%); and for the variable ≥ 5 years (OR = 1.41, 95% CI: 1.19 to 1.68, *p* = 0.000, I^2^ = 56.77%). Beeg’s test indicated no evidence of publication bias for variables of cessation of OC use <5 years, (*p* = 0.117) and ≥5 years (*p* = 0.117) before diagnosis. Data from Egger’s test indicate publication bias for both covariates: < 5 years (*p* = 0.022) and ≥ 5 years, *p* = 0.018 (Table 2). Multivariable meta-regression demonstrated a non-significant effect for both covariates: β = 0.76 (95% CI: 0.25 to 1.26), z = 2.96, *p* = 0.0031; β = −0.20 (95% CI: −0.51 to 0.11), z = −1.28, *p* = 0.2022.

In addition, the risk of developing ER−BrCa has been shown to be similar in premenopausal and postmenopausal women: OR = 1.05, 95% CI: 0.62 to 1.79, *p* = 0.850. No significant publication bias was detected by the Egger’s test (*p* = 0.258).

### 3.3. Effects of Oral Contraceptive Use on HER2-Positive Breast Cancer

**Figure 4:**
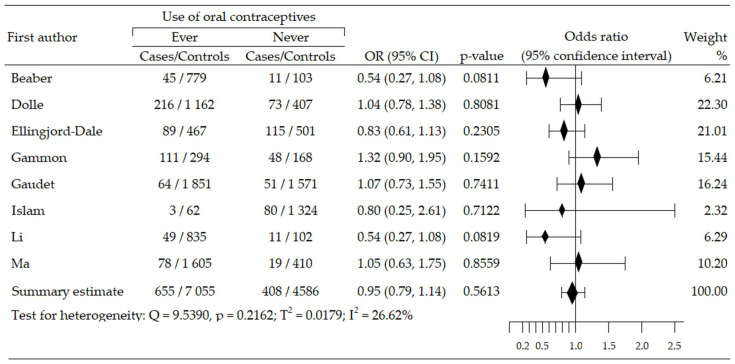
Forest plot and summary odds ratios on the association between risk of HER2+BrCa and ever-use of oral contraceptives. Note: black diamonds represent the effect sizes; the horizontal lines denote the 95% confidence interval.

Analysis of relationship between OCs use and risk of HER2-positive BrCa (HER2+ BrCa) subtype included eight trials, and was based on data from 12,704 participants (cases: 1063, control: 11,641) [45,48,49,50,54,55,58,61]. A statistically insignificant increase of cancer risk was reported in four studies [45,48,54,55], while in four trials, an insignificant risk reduction was seen [49,50,58,61]. The summary meta-analysis revealed that ever-use OC slightly decreased HER2+BrCa risk: OR = 0.95, 95% CI; 0.79 to 1.14, *p* = 0.561, I^2^ = 26.62% (Figure 4). Both tests, Begg’s and Egger’s, indicated no publication bias: *p* = 0.138 and *p* = 0.252, respectively (Table 2).

Assessment of dependencies between risk of HER2+BrCa subtype and duration of OCs use was done on data from five studies [45,48,49,54,61]. Taking OC for over five years was associated with a slightly increased risk of cancer: OR = 1.09, 95% CI: 0.88 to 1.35, *p* = 0.447, I^2^ = 19.01%. Results of the Egger’s and Begg’s tests did not reveal publication bias: *p* = 0.327 and *p* = 0.109, respectively. Moreover, the use of OC for less than five years was associated with a clear, albeit non-significant, reduction in the risk of HER+BrCa: OR = 0.88, 95% CI: 0.62 to 1.25, *p* = 0.480, I^2^ = 58.34%. Both tests were insignificant for publication bias: Begg’s test: *p* = 0.497, and Egger’s test: *p* = 0.855. Additionally, the risk of developing HER2+BrCa was slightly increased when using OC for more than five years, but the results were not statistically significant: OR = 1.14, 95% CI: 0.84 to 1.54, *p* = 0.412. No evidence of publication bias was recorded for the duration of OC use, according to Begg’s (*p* = 0.174) and Egger’s tests (*p* = 0.605) (Table 2). Multivariable meta-regression with covariates of duration of OC use demonstrated the absence of influence on risk of HER+BrCa subtype: β = −0.19, 95% CI: −0.59 to 0.21, z = −0.94, *p* = 0.349. 

Three studies evaluated dependence between period of last use of OC prior to diagnosis and occurrence of HER+BrCa subtype [45,48,54]. The period of less than five years was associated with a slightly higher, statistically insignificant risk of this subtype of breast cancer: OR = 1.09, 95% CI: 0.82 to 1.46, *p* = 0.555, I^2^ = 0.00%. In turn, discontinuation of OC intake ≥ 5 years before diagnosis indicated a higher, but non-significant HER+BrCa subtype risk: OR = 1.12, 95% CI: 0.90 to 1.40, *p* = 0.295, I^2^ = 0.00%. Results of Begg’s test and Egger’s test showed a lack of evidence of publication bias for the variable < 5 years: *p* = 0.602, and *p* = 0.485, respectively; the results for the variable ≥ 5 years also demonstrated absence of evidence of publication bias: *p* = 0.602 and *p* = 0.840, respectively. (Table 2). The result of multivariable meta-regression for period of last use OC prior to diagnosis did not confirm the impact of these covariates on the BrCa subtype: β = 0.03 (95% CI: −0.33 to 0.39), z = −0.16, *p* = 0.8705.

Moreover, the risk of breast cancer was slightly lower, although not statistically significant, in premenopausal women: OR = 0.79, 95% CI: 0.58 to 1.06, *p* = 0.116. No significant publication bias was detected by the Begg’s (*p* = 0.602) and Egger’s tests (*p* = 0.781).

### 3.4. Effects of Oral Contraceptive Use on Triplet-Negative Breast Cancer

**Figure 5:**
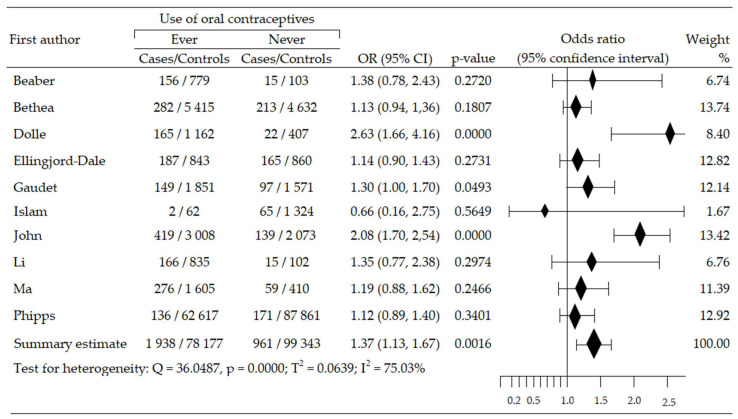
Forest plot and summary odds ratios on the association between risk of TNBrCa and ever-use of oral contraceptives. Note: black diamonds represent the effect sizes; the horizontal lines denote the 95% confidence interval.

We assessed the changes in the risk of triplet-negative breast cancer (TNBrCa) after the use of oral contraceptives based on data from ten case-control studies involving 180,419 women (including 2899 in case groups and 177,520 in control groups) [48,49,50,54,55,57,58,60,61,63]. Increased risk of TNBrCa development was reported in nine trials [48,49,50,54,55,57,60,61,63], including three studies that revealed statistical significance [48,55,63]. One study [58] reported a non-significant reduction in TNBrCa risk. Meta-analysis showed significant increase of TNBrCa risk in OC ever-use participants, compared with never-use: pooled OR = 1.37, 95% CI; 1.13 to 1.67, *p* = 0.002, I^2^ = 75.03% (Figure 5). Begg’s and Egger’s test did not reveal evidence of publication bias: *p* = 0.532 and *p* = 0.914 (Table 2).

The relationship between the age of initiation oral contraception and the risk of developing a TNBrCa was investigated based on three studies [54,57,61]. Age less than 25 years was associated with a significantly increased risk of this cancer subtype: OR = 1.27, 95% CI: 1.08 to 1.50, *p* = 0.005, I^2^ = 0.00%. Begg’s test (*p* = 0.117) and Egger’s test (*p* = 0.269) indicated no evidence of publication bias. In turn, starting of OC use at age ≥ 25 years was associated with slight increase in risk: OR = 1.03, 95% CI: 0.86 to 1.23, *p* = 0.758, I^2^ = 0.00%. Begg’s and Egger’s tests demonstrated the lack of publication bias: *p* = 0.602 and *p* = 0.966, respectively (Table 2). Multivariable meta-regression for age at start of contraceptive pill self-administration did not confirm the impact of these covariates on BrCa subtype: β = 0.03 (95% CI: −0.33 to 0.39), z = −0.16, *p* = 0.871.

Analysis of the impact of the duration of OC intake on TNBrCa risk was based on six studies [48,49,54,57,60,61]. OC use ≥ 5 years resulted in a significant increase in risk of developing this subtype cancer: OR = 1.46, 95% CI: 1.17 to 1.83, *p* = 0.001, I^2^ = 65.07%. Begg’s (*p* = 0.327) and Egger’s tests (*p* = 0.091) showed no evidence of publication bias. In contrast, taking OC < 5 years also led to an increased, albeit insignificant, risk of TNBrCa: OR = 1.16, 95% CI: 0.95 to 1.40, *p* = 0.146, I^2^ = 51.10%. Begg’s and Egger’s tests also demonstrated no evidence of publication bias: *p* = 0.189 and *p* = 0.199, respectively. Additionally, the comparison of these two groups showed that the use of OC for more than five years significantly increased risk of TNBrCa subtype: OR = 1.27, 95% CI: 1.12 to 1.42, *p* = 0.000. No significant publication bias was detected by the Begg’s (*p* = 0.851) and the Egger’s tests (*p* = 0.974),(Table 2). Multivariable meta-regression for the duration of OC indicated that the impact of the covariates was non-significant: β = −0.22 (95% CI: −0.52 to 0.08), z = −1.45, *p* = 0.1471.

Based on the results of three studies, we performed an analysis of the influence of years from the last use of OC before diagnosis on the risk of TNBrCa [48,54,60]. The results of the meta-analysis showed a statistically significant increase in risk (OR = 1.60, 0.95 CI: 1.01 to 2.53, *p* = 0.043, I^2^ = 71.09%) for last use < 5 years. The results of Begg’s test were inaccessible, while Egger’s test revealed no evidence of publication bias (*p* = 0.581). An increase in the risk of the cancer subtype was also observed, but was statistically insignificant regarding last OC use ≥ 5 years before diagnosis (OR = 1.41, 95% CI: 0.93 to 2.15, *p* = 0.107, I^2^ = 82.15%). Herein, the results of the Begg’s test were unavailable, while the Egger’s test indicated no evidence of publication bias (*p* = 0.248) (Table 2). Multivariable meta-regression did not record the significant influence of covariates of period from the last use of OC before diagnosis on TNBrCa risk: β = −0.12, 95% CI: −0.74 to 0.50, z = −0.39, *p* = 0.696.

In addition, the risk of breast cancer was slightly lower, although not statistically sig-nificant, in premenopausal women: OR = 0.90, 95% CI: 0.67 to 1.21, *p* = 0.489. No significant publication bias was detected by the Egger’s test (*p* = 0.279).

## 4. Discussion

Consistent with our results, the summary meta-analysis showed that ever-use of OC significantly increased the risk of TNBrCa, as well as of ER−BrCa. There was also a significant reduction in the risk of ER+BrCa and a slight reduction in the risk of HER2+BrCa after OC taking. This may indicate a protective OC effect in some molecular subtypes of BrCa. Furthermore, the initiation of OC use under the age of 25 years was associated with a significantly increased risk of TNBrCa and a non-significant reduction in the risk of ER+BrCa. In turn, the starting of OC use at the age of over 25 years resulted in a slight increase in the risk of TNBrCa and a slight reduction in the risk of ER+BrCa. 

Duration of OC use longer than five years was found to lead to a significant increase in risk of TNBrCa, and to an insignificant increase in risk of ER−BrCa and a slightly increased risk of HER2+ OC. In contrast, taking OC for less than five years led to an increased, albeit insignificant, risk of TNBrCa, as well as risks of ER−BrCa. Our work demonstrated that a shorter period of OC use was associated with a statistically significant reduction in the risk of ER+BrCa, and also with a clear, albeit non-significant, reduction in the risk of HER2+BrCa. 

The results of our study showed that the last use of OC in the period less than five years before diagnosis was associated with a statistically significant increase in risk of both TNBrCa and ER−BrCa, and with a slightly higher, statistically insignificant risk of HER2+BrCa. Moreover, we noted a non-significant reduction in risk of ER+BrCa. In turn, discontinuation of OC more than five years before diagnosis was associated with a statistically significant increase in ER−BrCa risk, and with a higher but non-significant risk of both TNBrCa and HER+BrCa, and a marginal increase in ER+BrCa. The only meta-analysis related to the above issue looked at the effect of oral hormonal contraception on the risk of TNBrCa. In this, Li et al. [64] showed that in women who took OC, there was a statistically significant increase in the incidence of TNBrCa: OR = 1.21, 95% CI: 1.01 to 1.41, *p* = 0.04.

In the previous article, we compiled, also using meta-analyzes, data from 79 case-control studies from 1960–2010. According to the results of the statistical analysis, there was an increased risk of BrCa with the use of OC before the first full-term pregnancy (OR = 1.14, 95% CI: 1.01 to 1.28, *p* = 0.036); as well as with the use of OC for more than five years (OR = 1.09, 95% CI: 1.01 to 1.18, *p* = 0.020). On the other hand, the use of OC before the age of 25 reduced the risk of BrCa: OR = 0.91, 95% CI: 0.83 to 1.00, *p* = 0.052 [34]. Our second meta-analysis included 42 studies published between 2009 and 2020. It turned out that the use of OC statistically significantly increased the risk of BrCa: OR = 1.15, 95% CI: 1.01 to 1.31, *p* = 0.036 [35].

The exact causes of the increased risk of ER-negative breast cancer with estrogen use have not yet been identified. The fact that estrogen promotes tumorogenesis of this subtype provides evidence that the presently observed effects occur via the influence of estrogens on the physiology of the tissues of the tumor-bearing host, rather than on the tumor cells themselves. One of the mechanisms by which the use of OC affects BrCa in women is the growth and angiogenesis of the tumor in the mammary gland caused by estrogen and ER positivity. A recent publication of Gupta et al. has proposed a second mechanism whereby estrogen promotes the growth of ER-negative breast cancer by systematically enhancing angiogenesis and stromal cell recruitment [65]. In the case of ER−BrCa and ER+BrCa, tumor growth may be favored by a mechanism that plays a major role in BrCa carcinogenesis and indicates that estrogen systematically increases vascular density and stromal cell recruitment [65]. The present observations indicate that estrogen increases the systemic capacity for angiogenesis, stromalization, and bone marrow cell recruitment, and that this mechanism is in part responsible for promoting tumorigenesis, including the growth of ER-negative tumors.

Great caution should be exercised in drawing final conclusions from our meta-analysis, as there were various limitations in conducting it. The value of the results may have been influenced by limiting the search results to works in English only. As a result of such a limitation, it was not possible to reach all the research related to the topic of our work. Secondly, retrospectively, self-reporting of use of oral contraceptives poses a risk of providing inaccurate data, and therefore the possibility of mistakes made in the recruitment to the control groups. Thirdly, there exists a possibility of errors occurring when cases are similar to the controls selected for the research, depending on the use of the OC. Fourthly, there is no available information on the type of oral contraceptive used, and also a possible source of bias is that the definition of “ever” for OC use is not unified, meaning that women are exposed to OC at various periods limited to the start and end of self-administration of pills. This may lead to misclassifications regarding the peak incidence of most cancers in old age with a long interval from last or first OC use, or the use of various hormone preparations in a woman’s life.

Additionally, a sensitivity analysis was performed to assess whether excluding any of the studies would significantly affect the meta-analysis result. The exclusion of any study did not affect the results of the meta-analysis of HER2 positive and triple negative breast cancer subtypes. However, in the case of the ER-positive breast cancer subtype, the exclusion of the Cotterchio [51], Gaudet [55] or Work [59] trials changed the result of the meta-analysis to statistically insignificant. In addition, when analyzing the ER-negative breast cancer subtype, the sensitivity analysis showed that excluding Bethea [60], Cotterchio [51], Dolle [48] or Sweeney [52] would change the meta-analysis result to statistically insignificant. Despite this, the authors did not decide to exclude the above studies, as in more than half of the cases this would increase the value of the standard error.

## 5. Conclusions

The results of our study suggest that the use of oral contraceptives has different effects on the risk of developing the various molecular breast cancer subtypes; however, given that associations between them are still poorly understood, further research is required.

## Figures and Tables

**Table 1:** Characteristics of selected cases-control studies assessing effects of oral contraceptive use on risk of molecular breast cancer subtypes.

Authors [Ref.] Year	Country	Study Year	Age Range	Breast Cancer Subtype; N (n%)	NOS Score
Gammon [45] 1999	USA	1990–1992	20–44	HER2+: 159 (69.8); CRL: 462 (63.6)	4
Althuis [46] 2003	USA	1990–1992	20–54	ER+: 769 (83.2); ER−: 510 (78.2); CRL: 1557 (80.0)	5
McCredie [47] 2003	Australia	1992–1999	<40	ER+: 357 (92.7); ER−: 261 (93.5); CRL: 564 (91.0)	6
Cotterchio [51] 2003	Canada	1995–1998	25–74	ER+: 1749 (44.0); ER−: 678 (57.4); CRL: 3346 (49.1)	6
Sweeney [52] 2007	USA	1999–2004	<35–65+	ER+: 1164 (62.0); ER−: 339 (71.1); CRL: 2513 (59.8)	7
Dey [53] 2009	India	2002–2005	<35–50+	ER+: 323 (1.9); ER−: 575 (3.0); CRL: 1208 (3.5)	6
Dolle [48] 2009	USA	1983–1992	21–45	ER+: 532 (74.1); ER−: 364 (83.8); HER2+: 289 (74.7); TN: 187 (88.2); CRL; 1569 (74.1)	8
Ma [54] 2010	USA	1994–1998	35–64	ER+: 765 (77.0); HER2+: 97 (80.4); TN: 335 (82.4); CRL: 2015 (79.7)	7
Gaudet [55] 2011	USA	1980–1982	20–56	ER+: 525 (54.3); HER2+: 115 (60.0); TN: 246 (65.9); CRL: 3422 (60.2)	7
Bao [56] 2011	China	1996–1998/2002–2005	20–70	ER+: 1719 (19.4); ER−: 960 (18.3); CRL: 3461 (20.0)	8
Phipps [57] 2011	USA	1993–1998	50–79	ER+: 2610 (40.5); TN: 307 (44.3); CRL: 150,478 (41.6)	7
Islam [58] 2012	Japan	2003–2005	20–79	ER+: 545 (3.9); HER2+: 91 (3.3); TN: 67 (3.0); CRL: 1386 (4.5)	5
Li [49] 2013	USA	2004–2010	20–44	ER+: 767 (88.1); HER2+: 60 (81.7); TN: 181 (91.7); CRL: 937 (89.1)	8
Beaber [50] 2014	USA	2004–2010	20–44	ER+: 730 (87.4), ER−: 246 (89.4); HER2+: 56 (80.4); TN: 171 (91.2); CRL: 882 (88.3)	8
Work [59] 2014	Multicenter ^a^	1995–2004	18–69	ER+: 2827 (72.7); ER−: 1085 (77.2); CRL: 2979 (78.3)	6
Bethea [60] 2015	USA	1993–2001	20–74	ER+: 1852 (53.5); ER−: 1044 (58.9); TN: 495 (60.0); CRL: 10,047 (53.9)	7
Ellingjord-Dale [61] 2017	Norway	2006–2014	50–69	ER+ 3904 (52.7); HER2+: 210 (45.2); TN: 365 (54.8); CRL: 21,651 (50.5)	8
Chollet-Hinton [62] 2017	USA	1996–2001	22–59	ER+: 162 (81.5); ER−: 169 (84.6); CRL: 1344 (86.8)	8
John [63] 2018	USA	1995–2002	35–79	TN: 558 (75.1); CRL: 5081 (59.2)	7

Abbreviations: CRL, control; ER−, estrogen receptor negative (regardless of their PR status); Er+, estrogen receptor positive (regardless of their PR/HER2 status); HER2+, human epidermal growth receptor 2-positive (ER−/PR−/HER2+); N, number of participants; n, percentage of ever OC use; TN, triple-negative breast cancer (ER−/PR−/HER2−). ^a^ USA, Canada, Australia, Korea.

**Table 2:** Pooled estimates of effect taking of oral contraceptives on subgroups breast cancer risk.

Subgroup Outcomes	n	OR	95% CI	*p*	I^2^ (%)	Begg’s Test			Egger’s Test			
						Tau-b	z	*p*	B0	95% CI	t	*p*
ER-positive breast cancer ^a^												
Oral contraceptives (OC) use [46,47,48,49,50,51,52,53,54,55,56,57,58,59,60,61,62]												
Ever	17	0.92	0.86 to 0.99	0.0256	66.59	−0.1167	−0.6303	0.5285	−0.7253	−2.4507 to 1.0000	−0.8960	0.3844
Never	17	Referent										
Age at first use of the OCs [54,57,61]												
≤25 years	3	0.93	0.72 to 1.19	0.5492	90.67	Inaccessible			−6.1506	−46.7092 to 34.4081	−1.9269	0.3048
>25 years	3	0.98	0.92 to 1.04	0.4697	0.00	−1.0000	−1.5667	0.1172	−0.7344	−0.8569 to −0.6119	−76.1858	0.0084
Duration of OCs use [46,48,49,51,52,54,57,59,60,61]												
≥5 years	10	0.91	0.82 to 1.02	0.1241	82.41	0.3333	1.0513	0.2931	−1.7854	−6.5313 to 2.9604	−0.8675	0.4109
<5 years	10	0.93	0.87 to 1.00	0.0421	53.09	−0.0222	−0.0894	0.9287	−0.0002	−3.1391 to 3.1387	−0.0001	0.9999
≥5 years/<5 years	10	0.99	0.91 to 1.08	0.7908	66.08	−0.4222	−1.6994	0.0892	−1.7506	−5.9584 to 2.4571	−0.9594	0.3654
Years since last use of OCs prior to diagnosis [46,48,52,54,60]												
<5 years	5	0.94	0.77 to 1.15	0.5553	66.34	−0.6667	−1.3587	0.1742	−1.4620	−12.1450 to 9.2210	−0.4355	0.6926
≥5 years	5	1.05	0.95 to 1.17	0.3463	49.58	−0.6667	−1.3587	0.1742	0.8787	−6.8687 to 8.6262	0.3610	0.7420
Menopausal status [53,55,56,58,59,61]												
Premenopausal	6	1.07	0.85 to 1.34	0.5745	93.14	Inaccessible			−5.0171	−18.9882 to 8.9539	−0.9970	0.3752
Postmenopausal	6	Referent										
ER-negative breast cancer ^b^												
Oral contraceptives use [46,47,48,50,51,52,53,56,59,60,62]												
Ever	11	1.20	1.03 to 1.40	0.0192	75.49	0.0222	0.0894	0.9287	0.1413	−3.2420 to 3.5247	0.0945	0.9268
Never	11	Referent										
Duration of OCs use [48,51,52,56,59,60]												
≥5 years	6	1.19	0.81 to 1.76	0.3733	94.39	Inaccessible			9.7359	−9.3757 to 28.8474	1.4144	0.2302
<5 years	6	1.14	0.93 to 1.40	0.2013	79.46	1.0000	2.0381	0.0415	3.4389	−7.6070 to 14.4847	0.8644	0.4361
≥5 years/<5 years	6	1.14	1.01. to 1.27	0.0306	36.52	1.0000	2.8180	0.0048	4.2529	3.1479 to 5.3579	10.6860	0.0004
Years since last use of OCs prior to diagnosis [46,48,52,60]												
<5 years	4	1.77	1.35 to 2.32	0.0000	68.44	1.0000	1.5667	0.1172	5.9849	2.0922 to 9.8775	6.6153	0.0221
≥5 years	4	1.41	1.19 to 1.68	0.0001	56.77	1.0000	1.5667	0.1172	3.9843	1.6504 to 6.3183	7.3452	0.0180
Menopausal status [53,56,59]												
Premenopausal	3	1.05	0.62 to 1.78	0.8499	96.82%	Inaccessible			−29.2517	−188.8490 to 130.3457	−2.3288	0.2582
Postmenopausal	3	Referent										
HER2-positive breast cancer												
Oral contraceptives use [45,48,49,50,54,55,58,61]												
Ever	8	0.95	0.79 to 1.14	0.5613	26.62	−0.4286	−1.4846	0.1376	−1.3610	−3.9893 to 1.2674	−1.2670	0.2521
Never	8	Referent										
Duration of OCs use [45,48,49,54,61]												
≥5 years	5	1.09	0.88 to 1.35	0.4465	19.01	−0.4000	−0.9798	0.3272	−2.9894	−7.1907 to 1.2120	−2.2644	0.1085
<5 years	5	0.88	0.62 to 1.25	0.4801	58.34	0.3333	0.6794	0.4969	0.5092	−7.6563 to 8.6748	0.1985	0.8554
≥5 years/<5 years	5	1.14	0.84 to 1.54	0.4123	54.09	−0.6667	−1.3587	0.1742	−1.5138	−9.8813 to 6.8537	−0.5758	0.6051
Years since last use of OCs prior to diagnosis [45,48,54]												
<5 years	3	1.09	0.82 to 1.46	0.5547	0.00	0.3333	0.5222	0.6015	0.9390	−10.4572 to 12.3352	1.0469	0.4854
≥5 years	3	1.12	0.90 to 1.40	0.2950	0.00	0.3333	0.5222	0.6015	0.6853	−33.1526 to 34.5232	0.2573	0.8396
Menopausal status [55,58,61]												
Premenopausal	3	0.79	0.58 to 1.06	0.1158	23.05	−0.3333	−0.5222	0.6015	−2.8198	−102.7123 to 97.0727	−0.3587	0.7808
Postmenopausal	3	Referent										
Triple-negative breast cancer												
Oral contraceptives use [48,49,50,54,55,57,58,60,61,63]												
Ever	10	1.37	1.13 to 1.67	0.0016	75.03	0.1667	0.6255	0.5316	0.1783	−3.5017 to 3.8582	0.1117	0.9138
Never	10	Referent										
Age at first use of the OC [8,11,17]												
≤25 years	3	1.27	1.08 to 1.50	0.0046	0.00	1.0000	1.5667	0.1172	5.1438	−24.2714 to 34.5591	2.2219	0.2692
>25 years	3	1.03	0.86 to 1.23	0.7578	0.00	−0.3333	−0.5222	0.6015	0.1671	−39.6385 to 39.9728	0.0534	0.9661
Duration of OCs used [7,8,11,13,16,17]												
≥5 years	6	1.46	1.17 to 1.83	0.0010	65.07	0.4000	0.9798	0.3272	3.6774	−0.9376 to 8.2924	2.2124	0.0914
<5 years	6	1.16	0.95 to 1.40	0.1458	51.10	0.4667	1.3151	0.1885	2.7372	−2.2003 to 7.6747	1.5392	0.1986
≥5 years/<5 years	6	1.26	1.12 to 1.42	0.0002	0.00	0.0667	0.1879	0.8510	0.1013	−8.0863 to 8.2888	0.0343	0.9743
Years since last use of OCs prior to diagnosis [7,8,16]												
<5 years	3	1.60	1.01 to 2.53	0.0433	71.09	Inaccessible			3.3617	−51.9058 to 58.6292	0.7729	0.5811
≥5 years	3	1.41	0.93 to 2.15	0.1068	82.15	Inaccessible			5.3614	−22.5766 to 33.2994	2.4384	0.2478
Menopausal status [55,58,61,63]												
Premenopausal	4	0.90	0.67 to 1.21	0.4892	70.15	Inaccessible			−2.9070	−11.4077 to 5.5937	−1.4714	0.2790
Postmenopausal	4	Referent										

Abbreviations: CI, confidence interval; ER, estrogen receptor; HER2, human epidermal growth factor 2; I^2^, coefficient of inconsistency; n, number of studies; OR, odds ratio; *p*, probability value. ^a^ regardless of their PR/HER2 status; ^b^ regardless of their PR status.

## Supplementary Materials

The following are available online at https://www.mdpi.com/article/10.3390/cancers14030574/s1, File S1: PRISMA 2020 Checklist.

## References

[1] Sung H., Ferlay J., Siegel R.L., Laversanne M., Soerjomataram I., Jemal A., Bray F. (2021). Global cancer statistics 2020: GLOBOCAN estimates of incidence and mortality worldwide for 36 cancers in 185 countries. CA Cancer J. Clin..

[2] Ferlay J., Colombet M., Soerjomataram I., Parkin D.M., Piñeros M., Znaor A., Bray F. (2021). Cancer statistics for the year 2020: An overview. Int. J. Cancer.

[3] Lynch H., Synder C., Wang S.M. (2015). Considerations for comprehensive assessment of genetic predisposition in familial breast cancer. Breast J..

[4] Bisgin A., Boga I., Yalav O., Sonmezler O., Tug Bozdogan S. (2019). BRCA mutation characteristics in a series of index cases of breast cancer selected independent of family history. Breast J..

[5] Yanagawa M., Ikemot K., Kawauchi S., Furuya T., Yamamoto S., Oka M., Oga A., Nagashima Y., Sasaki K. (2012). Luminal A and luminal B (HER2 negative) subtypes of breast cancer consist of a mixture of tumors with different genotype. BMC Res. Notes.

[6] Feng Y., Spezia M., Huang S., Yuan C., Zeng Z., Zhang L., Ji X., Liu W., Huang B., Luo W. (2018). Breast cancer development and progression: Risk factors, cancer stem cells, signaling pathways, genomics, and molecular pathogenesis. Genes Dis..

[7] Yersal O., Barutca S. (2014). Biological subtypes of breast cancer: Prognostic and therapeutic implications. World J. Clin. Oncol..

[8] Geyer F.C., Rodrigues D.N., Weigelt B., Reis-Filho J.S. (2012). Molecular classification of estrogen receptor-positive/luminal breast cancers. Adv. Anat. Pathol..

[9] Gao J.J., Swain S.M. (2018). Luminal A breast cancer and molecular assays: A review. Oncologist.

[10] Ades F., Zardavas D., Bozovic-Spasojevic I., Pugliano L., Fumagalli D., de Azambuja E., Viale G., Sotiriou C., Piccart M. (2014). Luminal B breast cancer: Molecular characterization, clinical management, and future perspectives. J. Clin. Oncol..

[11] Cheang M.C., Chia S.K., Voduc D., Gao D., Leung S., Snider J., Watson M., Davies S., Bernard P.S., Parker J.S. (2009). Ki67 index, HER2 status, and prognosis of patients with luminal B breast cancer. J. Natl. Cancer Inst..

[12] Inic Z., Zegarac M., Inic M., Markovic I., Kozomara Z., Djurisic I., Inic I., Pupic G., Jancic S. (2014). Difference between luminal A and luminal B subtypes according to Ki-67, tumor size, and progesterone receptor negativity providing prognostic information. Clin. Med. Insights Oncol..

[13] Putti T.P., Abd El-Rehim D.M., Rakha E.A., Paish C.E., Lee A.H.S., Pinder S.E., Ellis I.O. (2005). Estrogen receptor-negative breast carcinomas: A review of morphology and immunophenotypical analysis. Mod. Pathol..

[14] Wang C., Bai F., Zhang L.H., Scott A., Li E., Pei X.H. (2018). Estrogen promotes estrogen receptor negative BRCA1-deficient tumor initiation and progression. Breast Cancer Res..

[15] Senkus-Konefka E., Kunc M., Pęksa R., Łacko A., Radecka B., Braun M., Pikiel J., Litwiniuk M., Pogoda K., Cserni G. (2020). ER-/PgR+ breast cancer is a separate entity characterized by distinct phenotype: Comprehensive reevaluation of cases from Polish and Hungarian centers. J. Clin. Oncol..

[16] Ahmed S.S., Thike A.A., Zhang K., Lim J.C.T., Tan P.H. (2017). Clinicopathological characteristics of oestrogen receptor negative, progesterone receptor positive breast cancers: Re-evaluating subsets within this group. J. Clin. Pathol..

[17] Schroth W., Winter S., Büttner F., Goletz S., Faißt S., Brinkmann F., Saladores P., Heidemann E., Ott G., Gerteis A. (2016). Clinical outcome and global gene expression data support the existence of the estrogen receptor-negative/progesterone receptor-positive invasive breast cancer phenotype. Breast Cancer Res. Treat..

[18] Ha S.M., Chae E.Y., Cha J.H., Kim H.H., Shin H.J., Choi W.J. (2017). Association of BRCA Mutation Types, Imaging Features, and Pathologic Findings in Patients With Breast Cancer with BRCA1 and BRCA2 Mutations. AJR Am. J. Roentgenol..

[19] Hefti M.M., Hu R., Knoblauch N.W., Collins L.C., Haibe-Kains B., Tamimi R.M., Beck A.H. (2013). Estrogen receptor negative/progesterone receptor positive breast cancer is not a reproducible subtype. Breast Cancer Res..

[20] De Maeyer L., Van Limbergen E., De Nys K., Moerman P., Pochet N., Hendrickx W., Wildiers H., Paridaens R., Smeets A., Christiaens M.-R. (2008). Does estrogen receptor-negative/progesterone receptor-positive breast carcinoma exist?. J Clin Oncol..

[21] Foley N.M., Coll J.M., Lowery A.J., Hynes S.O., Kerin M.J., Sheehan M., Brodie C., Sweeney K.J. (2018). Re-Appraisal of Estrogen Receptor Negative/Progesterone Receptor Positive (ER−/PR+) Breast Cancer Phenotype: True Subtype or Technical Artefact?. Pathol. Oncol. Res..

[22] Iqbal N., Iqbal N. (2014). Human epidermal erowth eactor receptor 2 (HER2) in cancers: Overexpression and therapeutic implications. Mol. Biol. Int..

[23] Gabos Z., Sinha R., Hanson J., Chauhan N., Hugh J., Mackey J.R., Abdulkarim B. (2006). Prognostic significance of human epidermal growth factor receptor positivity for the development of brain metastasis after newly diagnosed breast cancer. J. Clin. Oncol..

[24] Figueroa-Magalhães M.C., Jelovac D., Connolly R.M., Wolff A.C. (2014). Treatment of HER2-positive breast cancer. Breast.

[25] Li X., Jing Yang J., Peng L., Sahin A.A., Huo L., Ward K.C., O'Regan R., Torres M.A., Meisel J.L. (2017). Triple-negative breast cancer has worse overall survival and cause-specific survival than non-triple-negative breast cancer. Breast Cancer Res. Treat..

[26] Yang C.-Q., Liu J., Zhao S.-Q., Zhu K., Gong Z.-Q., Xu R., Lu H.-M., Zhou R.-B., Zhao G., Yin D.-C. (2020). Recent treatment progress of triple negative breast cancer. Prog. Biophys. Mol. Biol..

[27] Rosato V., Bosetti C., Negri E., Talamini R., Dal Maso L., Malvezzi M., Falcini F., Montella M., La Vecchia C. (2014). Reproductive and hormonal factors, family history, and breast cancer according to the hormonal receptor status. Eur. J. Cancer Prev..

[28] Lambertini M., Santoro L., Del Mastro L., Nguyen B., Livraghi L., Ugolini D., Peccatori F.A., Azim H.A. (2016). Reproductive behaviors and risk of developing breast cancer according to tumor subtype: A systematic review and meta-analysis of epidemiological studies. Cancer Treat. Rev..

[29] Barnard M.E., Boeke C.E., Tamimi R.M. (2015). Established breast cancer risk factors and risk of intrinsic tumor subtypes. Biochim. Biophys. Acta.

[30] Eliassen A.H., Missmer S.A., Tworoger S.S., Spiegelman D., Barbieri R.L., Dowsett M., Hankinson S.E. (2006). Endogenous steroid hormone concentrations and risk of breast cancer among premenopausal women. J. Natl. Cancer Inst..

[31] Missmer S.A., Eliassen A.H., Barbieri R.L., Hankinson S.E. (2004). Endogenous estrogen, androgen, and progesterone concentrations and breast cancer risk among postmenopausal women. J. Natl. Cancer Inst..

[32] Mørch L.S., Skovlund C.W., Hannaford P.C., Iversen L., Fielding S., Lidegaard Ø. (2017). Contemporary Hormonal Contraception and the Risk of Breast Cancer. N. Engl. J. Med..

[33] Westhoff C.L., Pike M.C. (2018). Hormonal contraception and breast cancer. Am. J. Obstet. Gynecol..

[34] Kanadys W., Baranska A., Malm M., Błaszczuk A., Polz-Dacewicz M., Janiszewska M., Jedrych M. (2021). Use of oral contraceptives as a potential risk factor for breast cancer: A systematic review and meta-analysis of case-control studies up to 2010. Int. J. Environ. Res. Public Health.

[35] Barańska A., Błaszczuk A., Kanadys W., Malm M., Drop K., Polz-Dacewicz M. (2021). Oral Contraceptive Use and Breast Cancer Risk Assessment: A Systematic Review and Meta-Analysis of Case-Control Studies, 2009–2020. Cancers.

[36] Moher D., Shamseer L., Clarke M., Ghersi D., Liberati A., Petticrew M., Shekelle P., Stewart L.A. (2015). PRISMA-P Group. Preferred reporting items for systematic review and meta-analysis protocols (PRISMA-P) 2015 statement. Syst. Rev..

[37] Shamseer L., Moher D., Clarke M., Ghersi D., Liberati A., Petticrew M., Shekelle P., Stewart L.A. (2015). PRISMA-P Group. Preferred reporting items for systematic review and meta-analysis protocols (PRISMA-P) 2015: Elaboration and explanation. BMJ.

[38] Stang A. (2010). Critical evaluation of the Newcastle-Ottawa scale for the assessment of the quality of nonrandomized studies in metaanalyses. Eur. J. Epidemiol..

[39] DerSimonian R., Laird N. (1986). Meta-analysis in clinical trials. Control. Clin. Trials.

[40] Higgins J.P., Thompson S.G., Deeks J.J., Altman D.G. (2003). Measuring inconsistency in meta-analyses. BMJ.

[41] Begg C.B., Mazumdar M. (1994). Operating characteristics of a rank correlation test for publication bias. Biometrics.

[42] Egger M., Smith G.D., Schneider M., Minder C. (1997). Bias in meta-analysis detected by a simple, graphical test. BMJ.

[43] Duval S., Tweedie R. (2000). Trim and fill: A simple funnel-plot-based method of testing and adjusting for publication bias in meta-analysis. Biometrics.

[44] Baker W.L., White C.M., Cappelleri J.C., Kluger J., Coleman C.I. (2009). Health Outcomes, Policy, and Economics (HOPE) Collaborative Group. Understanding heterogeneity in meta-analysis: The role of meta-regression. Int. J. Clin. Pract..

[45] Gammon M.D., Hibshoosh H., Terry M.B., Bose S., Schoenberg J.B., Brinton L.A., Bernstein J.L., Thompson W.D. (1999). Oral contraceptive use and other risk factors in relation to HER-2/neu overexpression in breast cancer among young women. Cancer Epidemiol. Biomark. Prev..

[46] Althuis M.D., Brogan D.R., Coates R.J., Daling J.R., Gammon M.D., Malone K.E., Schoenberg J.B., Brinton L.A. (2003). Hormonal content and potency of oral contraceptives and breast cancer risk among young women. Br. J. Cancer..

[47] McCredie M.R., Dite G., Southey M.C., Venter D.J., Giles G., Hopper J.L. (2003). Risk factors for breast cancer in young women by oestrogen receptor and progesterone receptor status. Br. J. Cancer.

[48] Dolle J.M., Daling J.R., White E., Brinton L.A., Doody D.R., Porter P.L., Malone K.E. (2009). Risk factors for triple-negative breast cancer in women under the age of 45 years. Cancer Epidemiol. Biomark. Prev..

[49] Li C.I., Beaber E.F., Tang M.T.C., Porter P.L., Daling J.R., Malone K.E. (2013). Reproductive factors and risk of estrogen receptor positive, triple-negative, and HER2-neu overexpressing breast cancer among women 20-44 years of age. Breast Cancer Res. Treat..

[50] Beaber E.F., Malone K.E., Tang M.T., Barlow W.E., Porter P.L., Daling J.R., Li C.I. (2014). Oral contraceptives and breast cancer risk overall and by molecular subtype among young women. Cancer Epidemiol. Biomark. Prev..

[51] Cotterchio M., Kreiger N., Theis B., Sloan M., Bahl S. (2003). Hormonal factors and the risk of breast cancer according to estrogenand progesterone-receptor subgroup. Cancer Epidemiol. Biomark. Prev..

[52] Sweeney C., Giuliano A.R., Baumgartner K.B., Byers T., Herrick J.S., Edwards S.L., Slattery M.L. (2007). Oral, injected and implanted contraceptives and breast cancer risk among U.S. Hispanic and non-Hispanic white women. Int. J. Cancer..

[53] Dey S., Boffetta P., Mathews A., Brennan P., Soliman A., Mathew A. (2009). Risk factors according to estrogen receptor status of breast cancer patients in Trivandrum, South India. Int. J. Cancer..

[54] Ma H., Wang Y., Sullivan-Halley J., Weiss L., Marchbanks P.A., Spirtas R., Ursin G., Burkman R.T., Simon M.S., Malone K.E. (2010). Use of four biomarkers to evaluate the risk of breast cancer subtypes in the women's contraceptive and reproductive experiences study. Cancer Res..

[55] Gaudet M.M., Press M.F., Haile R.W., Lynch C.L., Glaser S.L., Schildkraut J., Gammon M.D., Thompson W.D., Bernstein J.L. (2011). Risk factors by molecular subtypes of breast cancer across a population-based study of women 56 years or younger. Breast Cancer Res. Treat..

[56] Bao P.P., Shu X.O., Gao Y.T., Zheng Y., Cai H., Deming S.L., Ruan Z.X., Su Y., Gu K., Lu W. (2011). Association of hormone-related characteristics and breast cancer risk by estrogen receptor/progesterone receptor status in the Shanghai Breast Cancer Study. Am. J. Epidemiol..

[57] Phipps A.I., Chlebowski R.T., Prentice R., McTiernan A., Wactawski-Wende J., Kuller L.H., Adams-Campbell L.L., Lane D., Stefanick M.L., Vitolins M. (2011). Reproductive history and oral contraceptive use in relation to risk of triple-negative breast cancer. J. Natl. Cancer Inst..

[58] Islam T., Matsuo K., Ito H., Hosono S., Watanabe M., Iwata H., Tajima K., Tanaka H. (2012). Reproductive and hormonal risk factors for luminal, HER2-overexpressing, and triple-negative breast cancer in Japanese women. Ann. Oncol..

[59] Work M.E., John E.M., Andrulis I.L., Knight J.A., Liao Y., Mulligan A.M., Southey M.C., Giles G.G., Dite G.S., Apicella C. (2014). Reproductive risk factors and oestrogen/progesterone receptor-negative breast cancer in the Breast Cancer Family Registry. Br. J. Cancer.

[60] Bethea T.N., Rosenberg L., Hong C.C., Troester M.A., Lunetta K.L., Bandera E.V., Schedin P., Kolonel L.N., Olshan A.F., Ambrosone C.B. (2015). A case-control analysis of oral contraceptive use and breast cancer subtypes in the African American Breast cancer epidemiology and risk consortium. Breast Cancer Res..

[61] Ellingjord-Dale M., Vos L., Tretli S., Hofvind S., Dos-Santos-Silva I., Ursin G. (2017). Parity, hormones and breast cancer subtypes—Results from a large nested case-control study in a national screening program. Breast Cancer Res..

[62] Chollet-Hinton L., Olshan A.F., Nichols H.B., Anders C.K., Lund J.L., Allott E.H., Bethea T.N., Hong C.C., Cohen SMKhoury T., Zirpoli G.R. (2017). Biology and etiology of young-onset breast cancers among premenopausal African American women: Results from the AMBER Consortium. Cancer Epidemiol. Biomark. Prev..

[63] John E.M., Hines L.M., Phipps A.I., Koo J., Longacre T.A., Ingles S.A., Baumgartner K.B., Slattery M.L., Wu A.H. (2018). Reproductive history, breast-feeding and risk of triple negative breast cancer: The Breast Cancer Etiology in Minorities (BEM) study. Int. J. Cancer..

[64] Li L., Zhong Y., Zhang H., Yu H., Huang Y., Li Z., Chen G., Hua X. (2017). Association between oral contraceptive use as a risk factor and triple-negative breast cancer: A systematic review and meta-analysis. Mol. Clin. Oncol..

[65] Gupta P.B., Proia D., Cingoz O., Weremowicz J., Naber S.P., Weinberg R.A., Kuperwasser C. (2007). Systemic stromal effects of estrogen promote the growth of estrogen receptor-negative cancers. Cancer Res..

